# Supplementary material for: Resistance of Leukemia Cells to 5-Azacytidine: Different Responses to the Same Induction Protocol
Source: Cancers (Basel). 2023 Jun 5;15(11):3063. doi: 10.3390/cancers15113063 (PMC10252867; doi:10.3390/cancers15113063)
Supplement: Supplementary file 1 [file cancers-15-03063-s001.zip › cancers-2405772-Supplementary.pdf]

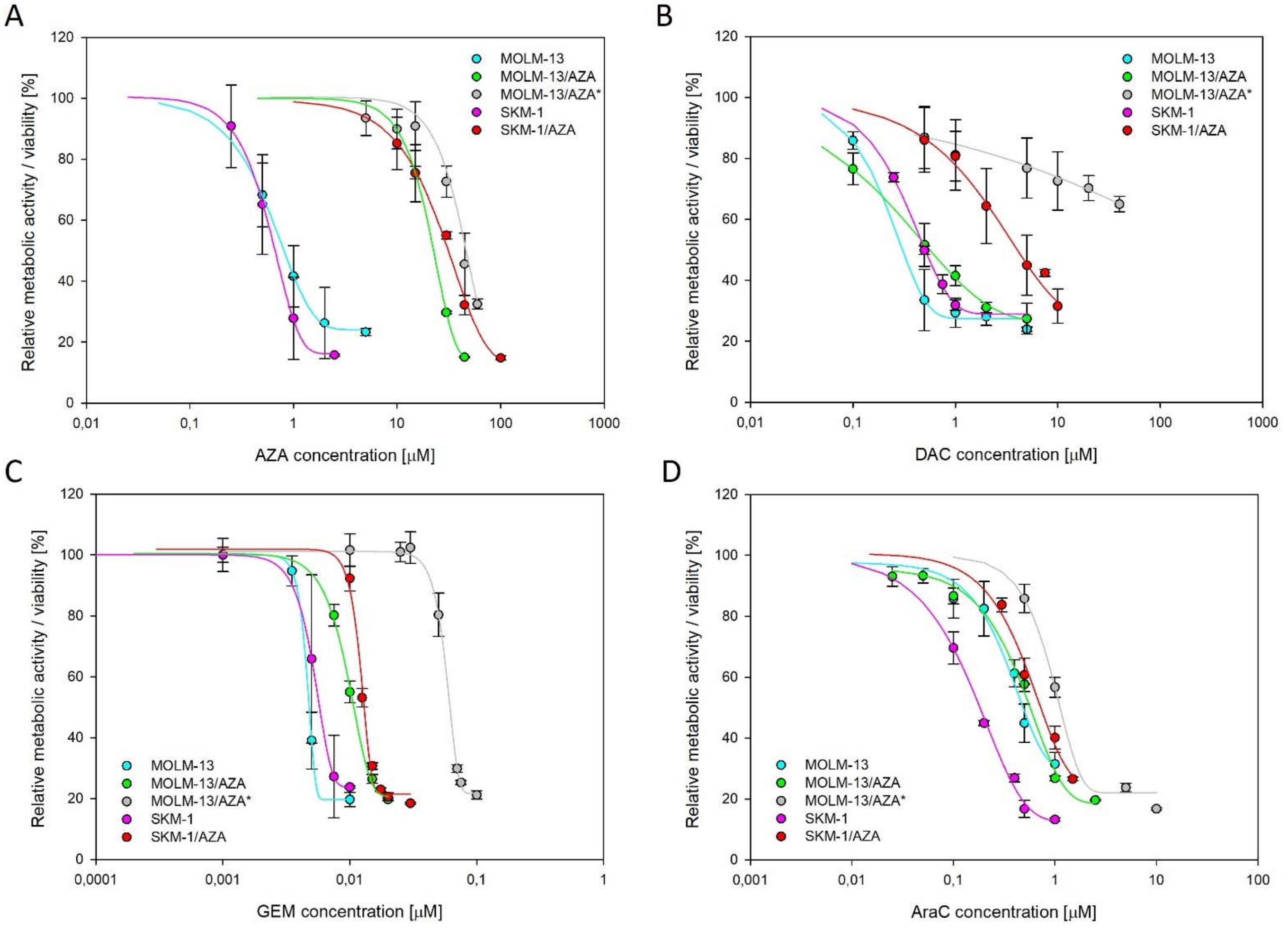

Figure S1. Dose response curves for the cell variants after 72 hours of cultivation with **A**) AZA and **B**) DAC added at various concentrations every 24 hours and after 48 hours of cultivation with **C**) GEM and **D**) AraC added once.

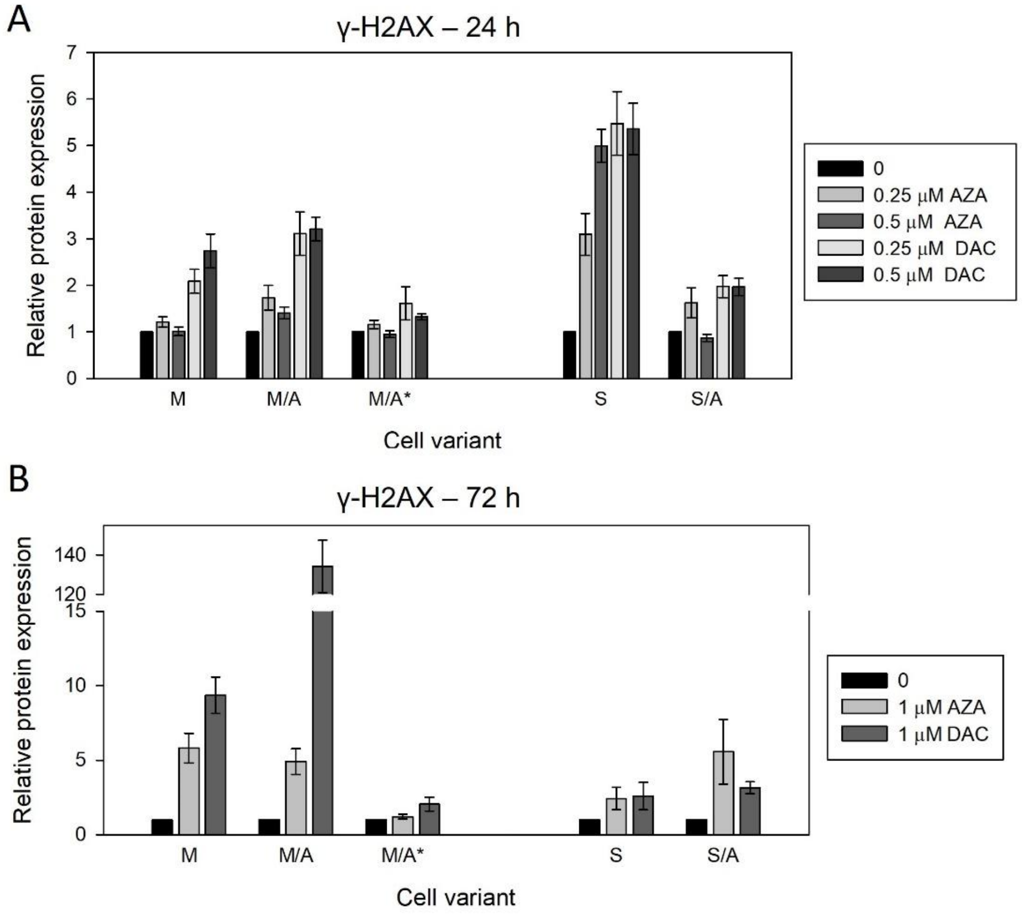

Figure S2. The optical densities of the γ-H2AX protein bands quantified by densitometry and summarized in the bar plots. The data were normalized to α-TUB, and the protein levels in the cells cultured without the HMAs were set as 1. The data are expressed as the mean ± SD of three independent measurements. **A**) 24-hour cultivation and **B**) 72-hour cultivation with either AZA or DAC.

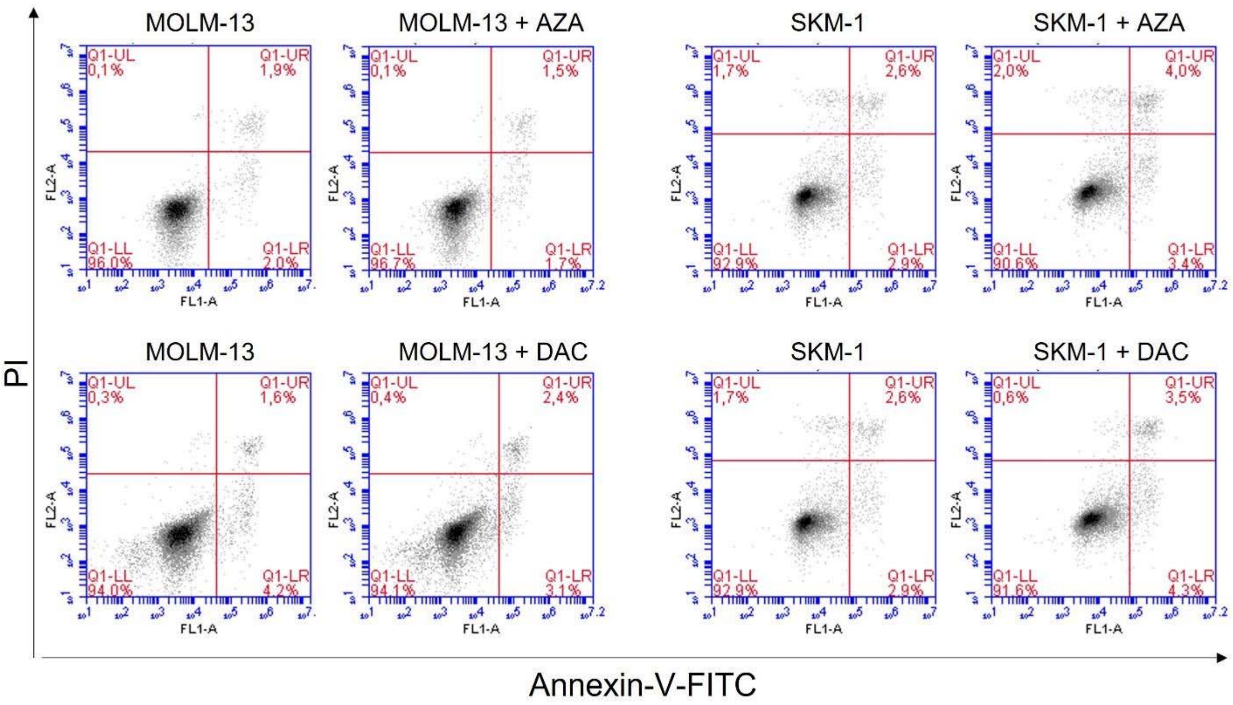

Figure S3. Representative dot plots of Annexin-V-FITC and PI assay for parental cell lines MOLM-13 and SKM-1 after 24-hour cultivation with or without 0.5 μM AZA or DAC.

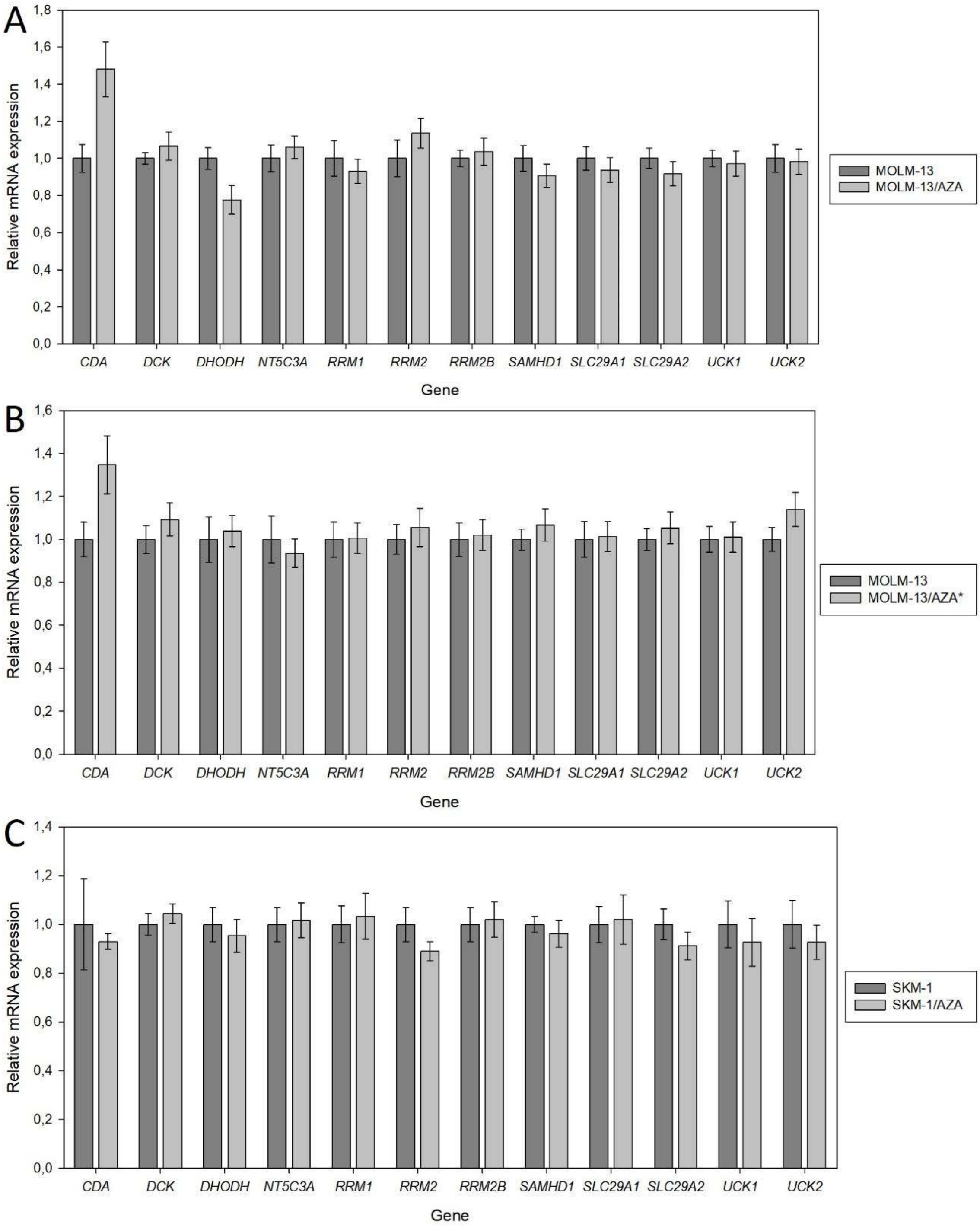

Figure S4. The optical densities of the PCR product bands were quantified and are summarized in bar plots. The data were normalized to *ACTB,* and the mRNA expression levels in parental cells were set as 1. The data are expressed as the mean ± SD of three independent measurements. **A**) Comparison of mRNA gene expression in the MOLM-13/AZA cell variant and parental MOLM-13 cell line. **B**) Comparison of mRNA gene expression in the MOLM-13/AZA* cell variant and parental MOLM-13 cell line. **C**) Comparison of mRNA gene expression in the SKM-1/AZA cell variant and parental SKM-1 cell line.

Table S1. Results of Pearson correlation analysis for correlation between data obtained by three different methods to determine the effect of TFN on each cell variant.

Cell variant	Correlation coefficient	P value
**Number of viable cells (CASY cell counter) vs. metabolic activity (MTS assay)**		
MOLM-13	0.924	$p \leq 0.05$
MOLM-13/AZA	0.929	$p \leq 0.05$
MOLM-13/AZA*	0.927	$p \leq 0.05$
SKM-1	0.942	$p \leq 0.02$
SKM-1/AZA	0.626	n.s. ($p > 0.1$)
**Number of viable cells (CASY cell counter) vs. proportion of viable cells (unstained by Annexin V and PI)**		
MOLM-13	0.923	$p \leq 0.05$
MOLM-13/AZA	0.844	m.s. ($p \leq 0.1$)
MOLM-13/AZA*	0.927	$p \leq 0.05$
SKM-1	0.964	$p \leq 0.01$
SKM-1/AZA	0.901	$p \leq 0.05$
**Metabolic activity (MTS assay) vs. proportion of viable cells (unstained by Annexin V and PI)**		
MOLM-13	0.997	$p \leq 0.001$
MOLM-13/AZA	0.587	n.s. ($p > 0.1$)
MOLM-13/AZA*	0.918	$p \leq 0.05$
SKM-1	0.994	$p \leq 0.001$
SKM-1/AZA	0.635	n.s. ($p > 0.1$)

n.s. – not significant, m.s. – marginally significant
