# Supplementary material for: Resistance of Leukemia Cells to 5-Azacytidine: Different Responses to the Same Induction Protocol
Source: Cancers (Basel). 2023 Jun 5;15(11):3063. doi: 10.3390/cancers15113063 (PMC10252867; doi:10.3390/cancers15113063)
Supplement: Supplementary file 1 [file cancers-15-03063-s001.zip › simonicova_et_al_membranes.pdf]

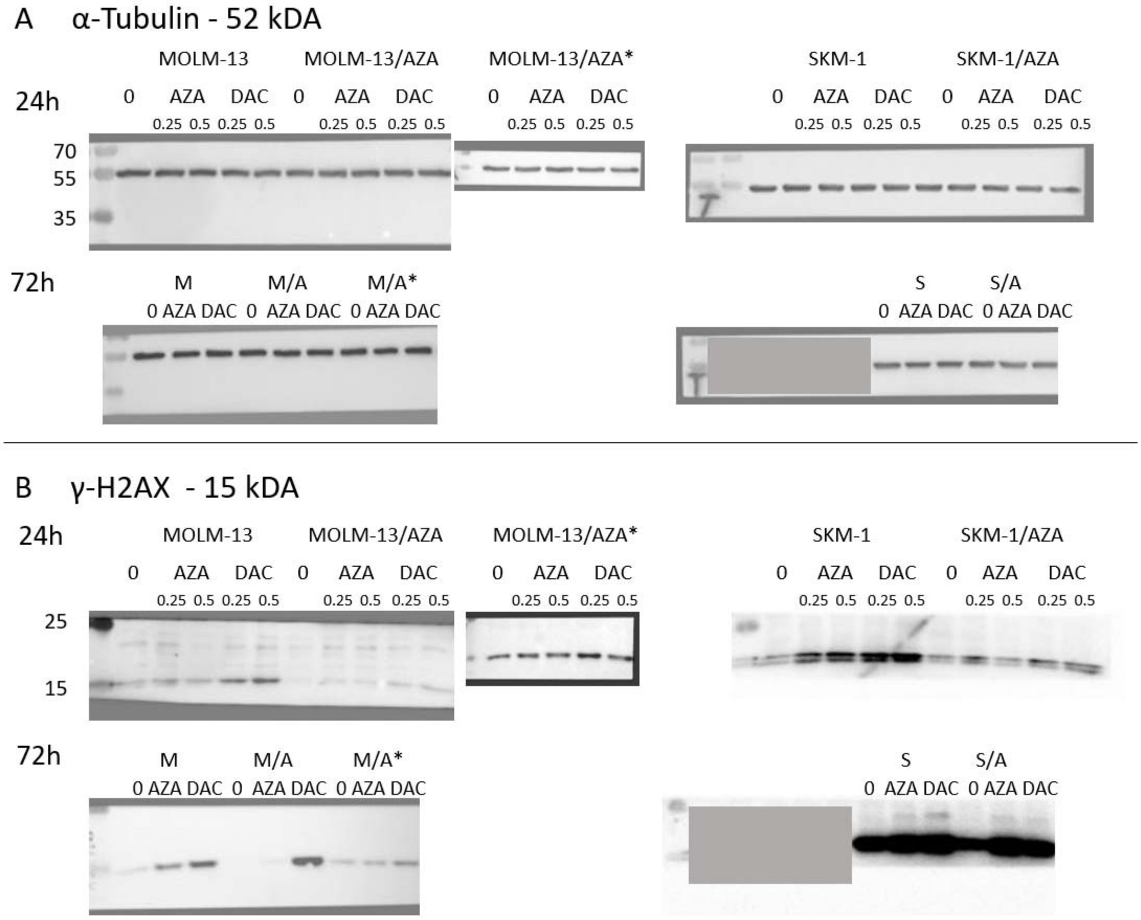


**Figure S5.** Uncropped membranes of **A**) α-Tubulin and **B**) γ-H2AX protein detection by Western blot (Figure 3B). Gray boxes – different cell variants, that are not the object of the current study and will be published in another article.

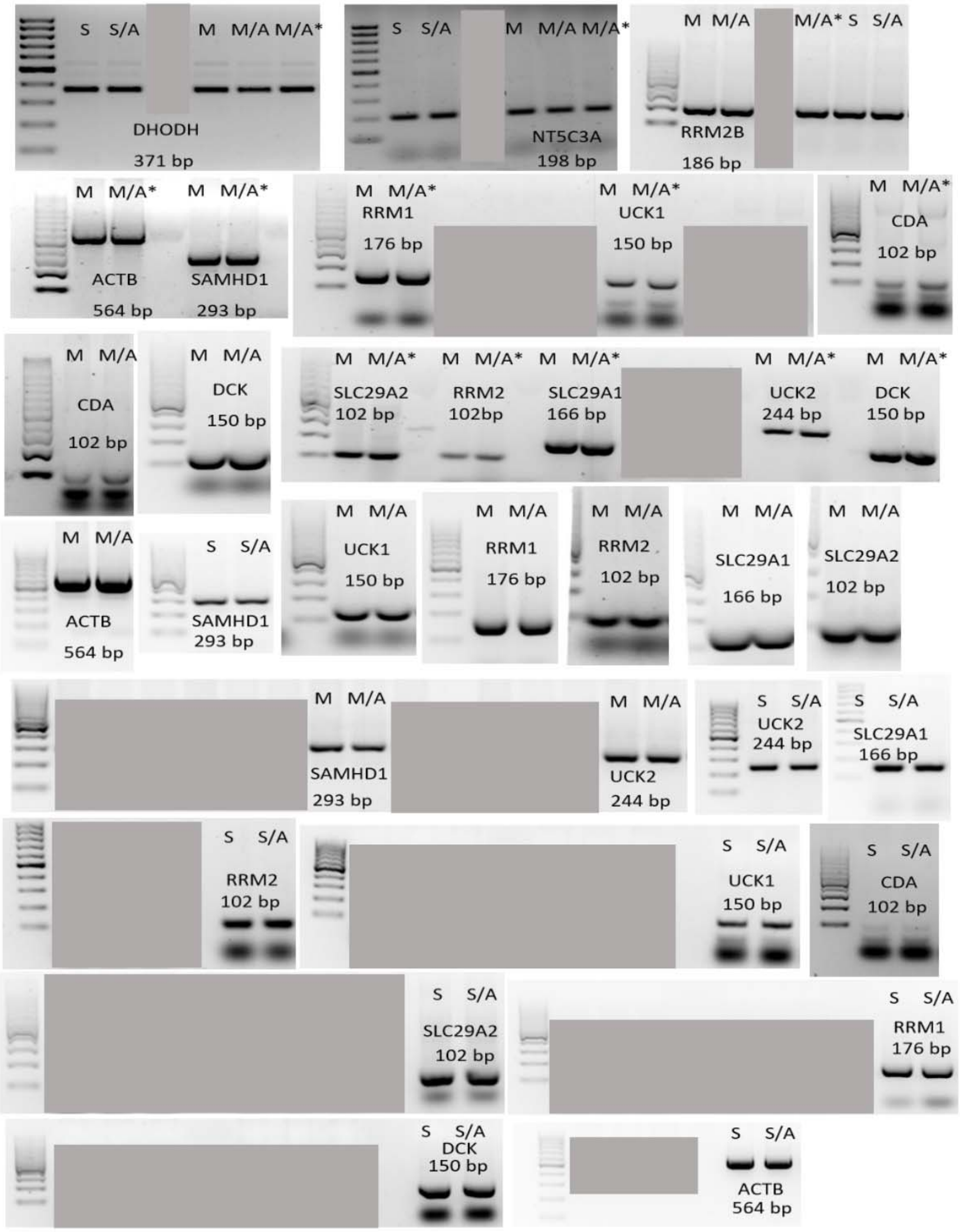


**Figure S6.** Agarose gels with electrophoretically separated RT-PCR products (Figure 4A). Gray boxes – different cell variants, that are not the object of the current study and will be published in another article.

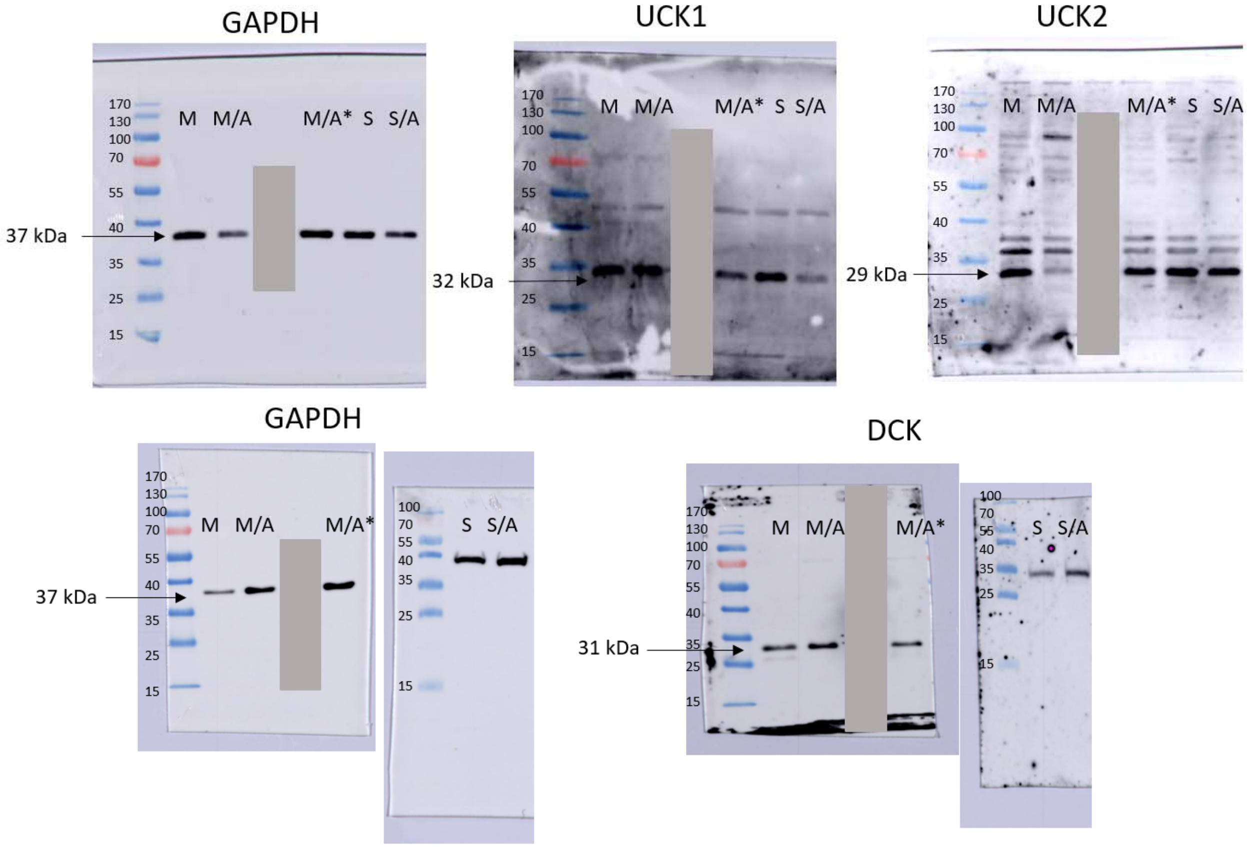


**Figure S7.** Uncropped membranes of GAPDH, UCK1, UCK2 and DCK protein detection by Western blot (Figure 4B). Gray boxes – different cell variants, that are not the object of the current study and will be published in another article.
